# Infection Probability Score, APACHE II and KARNOFSKY scoring systems as predictors of bloodstream infection onset in hematology-oncology patients

**DOI:** 10.1186/1471-2334-10-135

**Published:** 2010-05-26

**Authors:** Eleni Apostolopoulou, Vasilios Raftopoulos, Konstantinos Terzis, Ioannis Elefsiniotis

**Affiliations:** 1 University of Athens, Nursing Department, Athens, Greece; 2 Cyprus University of Technology, Nursing Department, Mediterranean Research Centre for Public Health and Quality of Care, Nicosia, Cyprus; 3 Halkida General Hospital, Halkida, Greece

## Abstract

**Background:**

Bloodstream Infections (BSIs) in neutropenic patients often cause considerable morbidity and mortality. Therefore, the surveillance and early identification of patients at high risk for developing BSIs might be useful for the development of preventive measures. The aim of the current study was to assess the predictive power of three scoring systems: Infection Probability Score (IPS), APACHE II and KARNOFSKY score for the onset of Bloodstream Infections in hematology-oncology patients.

**Methods:**

A total of 102 patients who were hospitalized for more than 48 hours in a hematology-oncology department in Athens, Greece between April 1^st ^and October 31^st ^2007 were included in the study. Data were collected by using an anonymous standardized recording form. Source materials included medical records, temperature charts, information from nursing and medical staff, and results on microbiological testing. Patients were followed daily until hospital discharge or death.

**Results:**

Among the 102 patients, Bloodstream Infections occurred in 17 (16.6%) patients. The incidence density of Bloodstream Infections was 7.74 per 1,000 patient-days or 21.99 per 1,000 patient-days at risk. The patients who developed a Bloodstream Infection were mainly females (p = 0.004), with twofold time mean length of hospital stay (p < 0.001), with fourfold time mean length of neutropenia (p < 0.001), with neutropenia < 500 (p < 0.001), suffered mainly from acute myeloid leukemia (p < 0.001), had been exposed to antibiotics (p = 0.045) and chemotherapy (p = 0.023), had a surgery (p = 0.048) and a Hickman catheter (p = 0.025) as compared to the patients without Bloodstream Infection. The best cut-off value of IPS for the prediction of a Bloodstream Infection was 10 with a sensitivity of 75% and specificity of 70.9%

**Conclusion:**

Between the three different prognostic scoring systems, Infection Probability Score had the best sensitivity in predicting Bloodstream Infections.

## Background

Bloodstream infections are an important cause of morbidity and mortality in immunocompromised populations [1]. Patients with hematologic malignancies such as acute leukemia, malignant lymphoma, and multiple myeloma are at increased risk for healthcare-associated bloodstream infections because of the intensive cytotoxic chemotherapy, which often renders them pancytopenic for long periods of time [2]. Several clinical trials showed improved survival with the use of immediate empirical broad-spectrum antimicrobial therapy at onset of fever, which has become the standard procedure [3,4]. Despite such an immediate approach, Bloodstream Infections (BSIs) in neutropenic patients often cause considerable morbidity and mortality. The high mortality is partly related to BSIs caused by microorganisms resistant to broad-spectrum antibiotics [5].

Laboratory-based strategies for the surveillance of bloodstream infections in the hematology department should be reported periodically, and used for longitudinal assessment [6]. Such data can then inform decisions about screening, prophylaxis and empirical therapy. If clinical data are also collected for surveillance purposes (e.g presence of CVC, risk factors for the development of bacteraemia), an understanding of the required resources and prevention strategies should also be available [7]. In patients with suspected bloodstream infections and new skin lesions, urgent biopsy of necrotizing hematologic lesions should be performed to aid in the diagnosis of disseminated mould infection. Non-culture based diagnosis techniques have been used for the early detection of fungaemia in hematology patients but are not recommended for routine use in the clinical practice [8]. In addition, the benefit of prophylaxis for the reduction of disease incidence must be weighed against the potential for the selection of resistant isolates [9]. Implementation of infection control practices is required, particularly for the control of transmission of multi-resistant organisms [10]. Surveillance may be justified in case of hospital outbreaks or periods of increasing endemicity, to facilitate the isolation and barrier precautions for the patients who are colonized or infected with multi-resistant organisms. For clusters or outbreaks of hospital infections, appropriate investigation, including environmental sampling, clinical screening and risk factor analysis may be required [11].

In Greece, the data concerning BSIs occurring in haematology/oncology units are sparse. Therefore, the surveillance and early identification of patients at high risk for developing BSIs might be useful for the development of preventive measures. The objective of this study was to assess the predictive power of three systems: Infection Probability Score (IPS), APACHE II and Karnofsky score for the onset of BSIs in hematology/oncology patients, which will provide a basis to design more effective guidelines for the prevention and treatment of these infections.

## Methods

A retrospective review of all records during a seven-month period (1/4/2007-30/10/2007) was conducted in the hematology-oncology unit of a general hospital in Athens, Greece. All patients who were hospitalized for more than 48 hours in the hematology-oncology unit were included in the analysis. The surveillance of nosocomial BSI was performed by the physician in charge of the patient and a specifically trained nurse. It included a daily clinical examination of the patients and daily reviewing of the patient's medical records, Kardex data, temperature charts, information from the nursing and the medical staff, and all the microbiological data of a positive blood result.

Data were collected by using an anonymous standardized case-record form. Patient data consisted of I) demographic characteristics: age, length of stay, outcome (survivors or non-survivors), II) risk factors: underlying malignancy, underlying diseases: Infection on admission, diabetes mellitus, short or long term presence of an indwelling central venous catheter (CVC), total parenteral nutrition, duration of neutropenia, exposure to chemotherapy, antibiotics or corticosteroid therapy and prior surgical procedure.

The diagnosis and the source of blood-stream infection were determined by the physician in charge and a specifically trained nurse, and were based on objective clinical evidence, microbiological data, clinical judgment and the definitions proposed by CDC. Blood cultures were performed in aerobic and anaerobic bottles and incubated in an automatic system. Bacteria were identified using standard microbiological methods. Antimicrobial therapy was given and adapted according to the susceptibility testing by the physician in charge of the patient. Patients were followed daily for BSI from admission until discharge or death. Patients were diagnosed with BSI on the date of the first positive result. The diagnosis of BSIs was based on definitions proposed by the Centers for Disease Control and Prevention [12].

### Ethical considerations

The research protocol was approved by the Ethics Committee of the Hospital in which the survey has been conducted. The anonymity and the confidentiality of the patients' data have been assured by the research team.

### Case definitions

Neutropenia was defined as an absolute blood neutrophil count less than 500/ml. Laboratory-confirmed bloodstream infection-LCBI: LCBI must meet at least one of the following criteria:

1. Patient has a recognized pathogen cultured from 1 or more blood cultures and organism cultured from blood is not related to an infection at another site.

2. Patient has at least one of the following signs or symptoms: fever (≥38°C) chills, or hypotension and signs and symptoms and positive laboratory results are not related to an infection at another site and common skin contaminant (ie, diphtheroids (Corynebacterium spp) Bacillus (not B anthracis) spp, Propionibacterium spp, coagulase-negative staphylococci (including S epidermidis) viridans group streptococci, Aerococcus spp, Micrococcus spp) is cultured from 2 or more blood cultures drawn on separate occasions.

The incidence rate was defined as the number of new cases of BSIs divided by the total number of patient-days and patient-days at risk (neutropenic days) in the population studied.

#### Instruments

The IPS is a simple scoring system that assesses the probability of infection in critically ill patients. It has been developed and validated in ICU [13]. IPS uses six simple and commonly used variables and ranges from 0-26 points (0-2 for temperature, 0-12 for heart rate, 0-1 for respiratory rate, 0-3 for white blood cell count, 0-6 for C-reactive protein, 0-2 for sequential organ failure assessment score [14].

APACHE II is a severity of disease classification system that uses a point score based on initial values of 12 routine physiologic measurements, age and previous health status, to provide a general measure of severity of disease. The total score in the APACHE II is 71, which includes the sum of physiological score, age score and chronic health evaluation [15].

The KARNOFSKY Performance Scale Index allows patients to be classified as to their functional impairment. The lower the KARNOFSKY score, the worse the survival for most serious illnesses [16]. The KARNOFSKY score runs from 100 to 0, where 100 is "perfect" health and 0 is death. Although the score has been described with intervals of 10, a practitioner may choose decimals if he or she feels a patient's situation holds somewhere between two marks [17]. Table 1 displays the score calculation of the three scales.

**Table 1:** Clinical characteristics of the patients

Characteristics	N = 102	%
Gender		

Male	53	52

Female	49	48

Hospital BSIs	17	17.3

Neutropenia	47	46.1

Deaths	8	7.8

**Hematology diseases**		

Acute myeloid leukemia	34	33.3

Acute lymphocytic leukemia	16	15.7

Multiple myeloma	15	14.7

Non Hodgkin's Lymphoma	14	13.7

Myelodysplastic syndrome	7	6.9

Aplastic anemia	6	5.9

Hodgkin's Lymphoma	4	3.9

Chronic Lymphocitic leukemia	4	3.9

Chronic myelogenous leukemia	2	2

Age (median)	53.30 ± 18.59, median 56 (Range 17-85)	

Length of stay(median)	21.54 ± 16.83, median 18 (Range 3-89)	

IPS score at admission	7.29 ± 6.41, median 6 (Range 0-21)	

IPS score for the first 3 days of hospitalization	6.92 ± 6.05, median 5.5 (Range 0-20)	

APACHE II score at admission	12.35 ± 0.44 median 12 (Range 5-32)	

APACHE II score for the first 3 days of hospitalization	11.79 ± 4.31 median 11 (Range 0-27)	

Karnofsky score in the admission	78.51 ± 14.89, median 80 (Range 40-100)	

### Data analysis

All of the items were coded and scored and the completed forms were included in the data analysis set. SPSS-17 (SPSS, Chicago, IL, US) was used to analyze the data. Continuous variables were compared using Student's *t-*test. The x^2 ^statistic or Fisher's Exact Test were used to compare categorical variables. All values are expressed as the mean ± SD or median for continuous variables and as a percentage of the group they were derived from categorical variables. ROC curve calculations have been performed by using MedCalc statistical software. Receiver operating characteristics (ROC) curves compared sensitivity and specificity of the three scoring systems (APACHE II, KARNOFSKY and IPS scores) over a wide range of cut-off values for predicting the onset of BSI. The area under the ROC is a convenient way of comparing classifiers. A random classifier has an area of 0.5, while an ideal one has an area of 1. The ROC curve was used to choose the best operating point. All p values of < 0.05 were considered statistically significant unless otherwise stated.

## Results

Over the seven-month period a total of 102 patients with 2,197 patient-days were admitted in a hematology/oncology unit in Athens-Greece and were prospectively evaluated. Among the participants, 53 (52%) were males and 49 (48%) were females. The baseline clinical characteristics of the patients are illustrated in Table 1. Patients had a mean age of 53.30 ± 18.59 years (median 56 years, range 17 - 85 years). The mean duration of neutropenia was significantly longer for patients with BSI than for patients without BSI (21.06 ± 8.33 vs 5.07 ± 8.32 days, p < 0.001). The majority of the patients (33.3%) suffered from acute myeloid leukemia (Tables 1 and 2).

**Table 2:** Characteristics of all patients with and without BSI

Variable	BSI N = 17	Non BSI N = 85	p value
Gender			
Male	3 (17.6%)	50 (57.7%)	0.004
Female	14 (82.3%)	35 (41.2%)	

Age	49.50 ± 14.62	54.01 ± 1.22	0.293

Length of stay	41.56 ± 20.05	17.81 ± 13.27	< 0.001

Duration of neutropenia	21.06 ± 8.33	5,07 ± 8,32	< 0.001

Karnofsky score	77.50 ± 16.93	81.40 ± 11.99	0.390

IPS score	11.06 ± 5.10	6.59 ± 6.41	0.010

Apache score	12.19 ± 4.60	12.38 ± 4.38	0.876

Neutropenia	16 (94.1%)	31 (36.5%)	< 0.001
**Underlying disease**			
Infection at admission	2 (11.7%)	23 (27.1%)	0.187
Diabetes	1 (5.9%)	6 (7.1%)	0.698

**Underling malignancy**			
Acute myeloid leukemia	13 (76.5%)	22 (25.9%)	< 0.001
Acute lymphocytic leukemia	4 (23.5%)	12 (14.1%)	0.221

Exposure to antibiotics	15 (88.2%)	61 (77.7%)	0.045

Exposure to chemotherapy	13 (76.5%)	44 (51.7%)	0.023

Corticosteroid therapy	3 (17.6%)	37 (43.5%)	0.057

Surgery procedure	3 (17.6%)	3 (3.5%)	0.048

Bronchoscopy	1 (5.9%)	3 (3.5%)	0.500

Gastroscopy	1 (5.9%)	6 (7.1%)	0.698

Parenteral nutrition	1 (5.9%)	1 (1.2%)	0.290

Bladder catheter	2 (11.7%)	7 (8.2%)	0.429

CVC	2 (11.7%)	2 (2.3%)	0.115

Hickman catheter	6 (35.3%)	11 (12.9%)	0.025

Peripheral venous catheter	12 (70.60%)	77 (90.6%)	0.119

Death	1 (5.9%)	7 (8.2%)	0.634

As it is displayed in Table 2, the mean APACHE II score did not differ statistically (p = 0.876) between those with a BSI and those without BSI. On the contrary IPS score was significantly (p = 0.010) higher in the patients with a BSI (11.06 ± 5.10) as opposed to those without BSI (6.59 ± 6.41).

The patients who developed a BSI were mainly females (p = 0.004), with twofold higher time for mean length of hospital stay (p < 0.001), with fourfold higher time for mean length of neutropenia (p < 0.001), with neutropenia < 500 (p < 0.001), suffered mainly from acute myeloid leukemia (p < 0.001), had been exposed to antibiotics (p = 0.045) and chemotherapy (p = 0.023), had a surgery (p = 0.048) and a Hickman catheter (p = 0.025) as compared to patients without BSI (Table 2).

The cumulative incidence of BSI was 16.7% and the incidence rate of BSI was 7.74 per 1,000 patient-days or 21.99 per 1,000 patient-days at risk. The most common isolates were gram-negative (44.4%) and gram positive (44.4%) microorganisms.

### Determination of the best cut-off point for the prediction of BSI onset

Table 3 illustrates the predictive values of the three scoring systems calculated at the best cut-off point according to the ROC curve. As can be seen, the IPS score system better predicts the onset of a BSI compared to the two other scoring systems. Discrimination power of IPS AUC was acceptable as opposed to APACHE II AUC and KARNOFSKY AUC that were poor. The best cut-off value of IPS for the prediction of a BSI was 10 with a sensitivity of 75% and a specificity of 70.9% (Table 3).

**Table 3:** Comparison of the three scoring systems in predicting the onset of BSI

Tool	Cut-off point	Sensitivity (%)	Specificity (%)	AUC	95% CI	PPV	NPV
IPS score	10	75	70.9	0.72	0.62-0.83	37.06	92.54
APACHE II score	15	25	86	0.49	0.39-0.59	28.98	83.38
KARNOFSKY score	70	43.7	76.7	0.55	0.45-0.65	42.88	87.08

PPV: Positive Predictive Value

NPV: Negative Predictive Value

Logistic regression analysis revealed that IPS was the only significant predictor of BSI onset (OR = 5.60; 95% CI = 1.66-18.88; p = 0.003) compared with APACHE II (OR = 0.71; 95% CI = 0.22-2.28; p = 0.546) and KARNOFSKY (OR = 0.39; 95% CI = 1.13-1.18; p = 0.121).

## Discussion and Conclusion

A retrospective study was conducted in order to examine the predictive power of three different scoring systems for BSI in a hematological unit. The literature regarding predictive power of IPS in hematology/oncology patients is sparse. Most of the information about the epidemiology of BSIs and outcome is extrapolated from studies of hospitalized patients in general hospitals or from studies of patients in University hospitals [18] or, more specifically, from studies of neutropenic cancer patients [19]. In our study, the incidence density of BSIs (21.99 per 1,000 patient-days at risk) was higher than that reported by previous studies (11.2 per 1000 patient-days at risk) [20]. Furthermore in the current study the crude mortality (5.9%) was lower than that reported elsewhere [21]. One of the major problems associated with comparison studies, is the difficulty in interpretation caused by variability in study designs and efficacy or outcome measures [22]. Furthermore, many patients with neutropenia have complex medical profiles, leading to increased physician concern and a tendency to modify regimens during the treatment course of febrile episodes. Such modifications generally are made in a setting of inadequate information or lack of definitive diagnosis. It has been estimated that empiric regimens may be modified in 40% to 60% of cases [23].

Furthermore, we found that the most common isolates were gram-negative (44.4%) and gram positive (44.4%) microorganisms. Our findings are very similar to those reported in other studies [24]. It is noteworthy that a considerable shift in the spectrum of pathogens isolated from blood cultures of febrile neutropenic cancer patients in the USA has emerged since 1995, with Gram-positive cocci increasing from 62% to 76% of isolates [21], Gram-negative bacilli declining from 21.5% to 14% and fungi declining from 15 to 8%. Based on consecutive clinical trials conducted by the European Organization on the Research and treatment of cancer (EORTC) between 1973 and 2000, a shift toward the predominance of Cram-positive bacteria has also been reported however, during the most recent time period (1998 - 2000), Gram- positive and Gram-negative isolates have been involved in the same magnitude [24].

A number of epidemiological factors may have contributed to this changing pattern. Firstly, this period corresponds to the uptake of widespread empiric antibiotic therapy for febrile neutropenia especially fluroquinolones and third/fourth generation cephalosporines), with resulting selection pressure for gram-positive bacteria [25]. Secondly, the use of more intensive treatments with more severe oral mucositis may provide a frequent portal of entry for gram-positive organisms. Thirdly, increased utility of medium to long-term venous access devises for patients with malignancy may increase gram-positive blood stream infections (particularly staphylococcal infections [26]. Finally, local infection control practices may impact upon the number of infections and spectrum of causative organisms. Further investigation of the data of patients with BSIs showed that most patients had received third/fourth generation cephalosporines (53%) and quinolones (35.3%), and they had central venous (11%) and Hickman catheters. Our findings highlight that the ongoing co-operation between haematologists, oncologists and infectious disease specialists is important to detect trends in epidemiology, which can be used to design empirical antibiotic regimens and guide infection control policies [27].

The mean IPS score was higher in patients with a BSI. This is a common finding in the two other researches supporting the discriminative adequacy of the tool [13,28]. The two previous studies have confirmed the predictive power of the IPS for the onset of a Healthcare Associated Infection (HCAI). The current study extends the use of IPS as a predictor of the onset of a BSI. Additionally, the IPS separated infected from non-infected patients already on the first day of evaluation [28]. This finding enables the therapeutic team to early detect the infected patients, as well as to avoid the administration of unnecessary antibiotics to the patients that are unlikely considered to be infected. Further studies with a bigger sample would be useful to give more evidence to this argument.

Between the three different prognostic scoring systems, IPS had the best sensitivity in predicting BSI. It is worth noting that IPS as a risk factor for BSIs in cancer patients has never being studied before. A previous study has used IPS to predict the need for mechanical ventilation and the duration of mechanical ventilation [29] and for the prediction of HCAI in the ICU patients [28]. In a previous study [28] the cutoff point for the prediction of a HAI was 14 with a positive predictive value of 53.6% and a negative predictive value of 89.5%. In our study the cutoff point for the prediction of a BSI was 10 with a positive predictive value of 37% and a negative predictive value of 92.5%. The patients were considered as unlikely to be infected with a BSI if they had a score < 10 points. An IPS score >10 represents an argument against a BSI onset in oncology-hematology patients and should be considered as a complementary tool for the early detection of a BSI. In the present study the best cut-off value of IPS for the prediction of a BSI was 10 as opposed to 14 that has been previously reported from Martini *et al*. [28] and Peres-Bota [13]. One possible explanation is that the present research has been conducted in a sample of hematology/oncology patients who had a different nosological profile compared to the ICU patients. Furthermore Martini *et al*. [28] have used a priori the cut-off value 14 without testing its sensitivity and specificity as opposed to our study that has used ROC analysis to find the best cut-off value. A further research is needed to compare IPS scores between several categories of patients. A multi-centered study would be useful to test the reliability of the tool in patients with a similar pathology.

The AUC value of IPS is associated with a good test as it is above 0.70 with means that the probability that the patients will be correctly classified as an infected person is 72%. It may be considered that a bigger sample of bloodstream infected person would help us to make safer conclusions. Furthermore it could be argued that the tool is more sensitive in predicting the onset of a HAI compared with the onset of a BSI. In conclusion the calculation of the IPS score is simple and the variables used can be obtained routinely, without additional costs. Additionally it is easy to be used from the clinicians.

To reduce the risk of BSIs and associated mortality in the hematology population, a number of strategies may be implemented including the routine use of IPS. Early identification of a changing spectrum and antimicrobial sensitivity of isolates should always be monitored. Frequently, a multi-disciplinary approach is recommended, to facilitate certain interventions such as typing of isolates, ward cleaning and structural modification of clinical areas. Guidelines for empirical antibiotic therapy for febrile neutropenia must be based upon the risk of infection in specific hematology patients and the local epidemiology and susceptibility of infecting isolates.

## Competing interests

The authors declare that they have no competing interests.

## Authors' contributions

EA wrote the manuscript and was involved in the study design. VR wrote the manuscript, analyzed the data, interpreted the results, revised the manuscript and was involved in the study design. KT collected the data, and contributed to the literature review. IE read the manuscript. All the authors read and approved the final manuscript.

## Pre-publication history

The pre-publication history for this paper can be accessed here:

http://www.biomedcentral.com/1471-2334/10/135/prepub
